# Social Interactions through the Eyes of Macaques and Humans

**DOI:** 10.1371/journal.pone.0056437

**Published:** 2013-02-15

**Authors:** Richard McFarland, Hettie Roebuck, Yin Yan, Bonaventura Majolo, Wu Li, Kun Guo

**Affiliations:** 1 School of Psychology, University of Lincoln, Lincoln, United Kingdom; 2 School of Physiology, University of the Witwatersrand, Johannesburg, South Africa; 3 State Key Laboratory of Cognitive Neuroscience and Learning, Beijing Normal University, Beijing, China; University of Jyväskylä, Finland

## Abstract

Group-living primates frequently interact with each other to maintain social bonds as well as to compete for valuable resources. Observing such social interactions between group members provides individuals with essential information (e.g. on the fighting ability or altruistic attitude of group companions) to guide their social tactics and choice of social partners. This process requires individuals to selectively attend to the most informative content within a social scene. It is unclear how non-human primates allocate attention to social interactions in different contexts, and whether they share similar patterns of social attention to humans. Here we compared the gaze behaviour of rhesus macaques and humans when free-viewing the same set of naturalistic images. The images contained positive or negative social interactions between two conspecifics of different phylogenetic distance from the observer; i.e. affiliation or aggression exchanged by two humans, rhesus macaques, Barbary macaques, baboons or lions. Monkeys directed a variable amount of gaze at the two conspecific individuals in the images according to their roles in the interaction (i.e. giver or receiver of affiliation/aggression). Their gaze distribution to non-conspecific individuals was systematically varied according to the viewed species and the nature of interactions, suggesting a contribution of both prior experience and innate bias in guiding social attention. Furthermore, the monkeys’ gaze behavior was qualitatively similar to that of humans, especially when viewing negative interactions. Detailed analysis revealed that both species directed more gaze at the face than the body region when inspecting individuals, and attended more to the body region in negative than in positive social interactions. Our study suggests that monkeys and humans share a similar pattern of role-sensitive, species- and context-dependent social attention, implying a homologous cognitive mechanism of social attention between rhesus macaques and humans.

## Introduction

In group-living mammal and bird species (e.g. primates, dolphins and ravens), individuals display different social tactics and select social partners based on their past interactions with other group members [1]. By observing the social interactions of their group companions, animals can gather a significant amount of information about an individual, such as its dominance position, fighting ability and response to affiliative social solicitations [2]–[4]. Such acquired information is considered fundamental to an individual’s decision to choose social partners, form alliances or avoid aggressive individuals [1], [3]. Therefore, attending to social interactions exchanged by other group members has fitness consequences, as it affects an individual’s behaviour and social tactics. Despite this, we know very little about the visual cues used by animals to acquire information from relevant social interactions, and whether social attention processes differ depending on the type of interaction observed and the individuals involved.

Human eye tracking studies have clearly demonstrated that active scene exploration is associated with a series of saccades to direct our fixation and attention toward local regions that are informative or important to us. The preferred regions within a scene are often inspected earlier and attract more fixations and longer viewing time. Therefore, gaze distribution provides a real-time behavioural index of ongoing perceptual and cognitive processing, and is reflective of our attention, motivation and preference; especially when exploring scenes of high ecological validity [5], [6]. With visual stimuli in simplistic social context (e.g. a face or human figure presented in isolation), previous studies have demonstrated that the gaze behaviour of monkeys is strikingly similar to that of humans during free-viewing. For instance, when presented with a face picture, both species often demonstrate a face-specific natural gaze bias towards the left hemi-face [7], and direct a disproportionate amount of fixations to the socially informative local facial features (i.e. eyes, nose and mouth region); with a strong preference towards the eyes [8]–[15]. It seems that monkeys and humans are broadly tuned to the same local visual cues in the processing of simplistic social scenes, suggesting a close evolutionary connection in the organisation of their visual system, as well as the visual and social behaviour observed in the two species. The simplified scenes used in these studies, however, do not represent naturalistic social interactions of which primates have prior experience. This issue can limit and potentially bias our understanding of social attention in primates. For instance, presenting a face or an animal in isolation, or in an artificially structured image (e.g. two animals combined together in a single scene), is clearly less ecologically-relevant than an image depicting a real-life social interaction. Therefore, gaze preference to certain facial or body regions embedded in these images may not necessarily be a true representation of primates’ social attention under natural conditions.

A few human eye tracking studies have examined gaze allocation in free-viewing of social scenes containing multiple people. Overall, the observers tend to spend the majority of their time looking back and forth between individuals in the scenes, and their attention is biased towards the faces, and in particular the eyes [16]–[18]. Furthermore, gaze allocation towards individual people or body regions is influenced by social action, content and context. For instance, the viewers tended to look more, and for longer, at the eyes of the face as the number of people in the scene increased, especially when these people were active [17]. In comparison with other people in the scene, individuals perceived as holding a higher social status [19], or were talking [18], tended to attract more fixations.

Like humans, monkeys also seem to respond to the content of biologically relevant social scenes [20]–[23]. When viewing video clips they tend to gaze towards individual people or animals in the scene, and look more often at their faces [20], [21]. When watching video clips of conspecifics, rhesus macaques altered their gaze and head orientation (i.e. aversion or following) according to their interest in, and actions of, monkeys within the video [22]. Moreover, increased visual attention and pupil diameter (i.e. sympathetic arousal) has been observed in rhesus macaques watching social, compared to non-social videos [23]. Finally, in a study comparing the pattern of visual attention of humans and rhesus macaques watching video clips [21], the gaze behaviour of both species was correlated with the biological relevance of the stimuli, driven by both content- and context-specific social cues.

Although these recent eye-tracking studies have examined human and monkey gaze behaviour when viewing biologically relevant social videos often containing more than one individual [22], [23], there has been no systematic investigation and direct comparison of how humans and non-human primates allocate their attention to different individuals in scenes of different social context (e.g. affiliation or aggression), and how this is affected by the viewed species.

An individual’s attention to a social interaction depends on how biologically relevant the interaction is, which ultimately is affected by phylogeny and/or prior experience. Various behavioural patterns, such as affiliation and aggression, share some homologous characteristics across different species [3], [24] and therefore might attract similar patterns of social attention and gaze behaviour. However, if phylogeny plays a dominant role in guiding social attention, we may expect that an individual’s gaze behaviour for viewing conspecifics and non-conspecifics would become increasingly different with the increasing phylogenetic distance between the two viewed species. For example, rhesus macaques and Barbary macaques (*M. sylvanus*) share similar body postures and facial displays for aggressive, submissive or affiliative exchanges - due to their phylogenetic relatedness [24]. Therefore, the gaze pattern of rhesus monkeys toward social interactions of their conspecifics is likely to be more similar to those patterns observed when viewing social interactions of Barbary macaques, when compared to those observed when viewing lions. Alternatively, if past experience shapes an individual’s viewing behaviour to social interaction scenes, similar gaze patterns may appear when observing the same type of social interaction in conspecifics and in familiar non-conspecifics (e.g. humans for laboratory-raised rhesus monkeys), regardless of the phylogenetic relatedness of the two viewed species.

In this study we aimed to compare gaze distribution in viewing naturalistic photographic images of social interactions in five different species (rhesus macaque, Barbary macaque, baboon: *Papio spp.*, lion: *Panthera leo*, and humans, respectively). We examined how rhesus macaques and humans distribute visual attention to different social interactions (i.e. affiliation or aggression) between their conspecifics and between individuals of other species within a range of phylogenetic distance and differing in terms of prior experience (i.e. familiar or unfamiliar species). Given laboratory-raised monkeys have limited social contact with non-human non-conspecifics, this comparison would help us to understand to what extent the social attention to conspecific interactions is a learned or innate trait. Furthermore, as rhesus monkeys are the most commonly used animal model of human perceptual and cognitive processes, it is essential to understand how close these two species are in their processing of social interaction scenes.

All images used in this study represented either a positive (i.e. affiliation) or negative (i.e. aggression) social context, and consisted of two ‘social roles’, a giver and a receiver of the relevant behaviour. Affiliative behaviours are pro-social behaviours that bring two or more individuals in to physical contact. We chose grooming exchange to represent positive social interaction, as it is the most common form of affiliation in primates [25]. Moreover, contact affiliation can also be observed across a range of mammals, including lions [26]. Aggression (i.e. one animal chasing and/or attacking another) was chosen to represent negative social interaction, as aggressive displays often share similar features across mammal species and can be easily recognized by (at least human) viewers. With these images representing naturalistic social interactions, we intended to address the following questions: 1) Can rhesus macaques differentiate between conspecific individuals based on their roles in different social interactions? That is, do they spend proportionally more time looking at the giver or receiver in positive and negative social interactions of other rhesus macaques? 2) Do rhesus macaques generalise this gaze behaviour across non-conspecific social interactions (i.e. Barbary macaques, baboons, lions and humans) and is this affected by phylogenetic distance between their own and other species, or by prior experience? 3) Which local regions (e.g. head, face or body) do rhesus macaques attend more frequently to extract diagnostic visual cues for processing scene contents? 4) Do rhesus macaques share similar viewing behaviour to humans in the viewing of the same social scenes?

## Materials and Methods

### Subjects

Four male adult rhesus macaques (5–9 kg, 5–9 years old) participated in this study. The animal experiments were conducted at Beijing Normal University. Ethical approval was granted by the Institutional Animal Care and Use Committee of Beijing Normal University, with all procedures in compliance with the National Institutes of Health Guide for the Care and Use of Laboratory Animals. The monkeys were born in captivity and socially housed indoors. Before the experiment, a custom-made biocompatible titanium head restraint (a small post on a cross-shaped pedestal with screw holes) was attached to the animal’s skull with titanium bone screws under aseptic conditions. The animals were prepared under general anesthesia induced with ketamine (10 mg/kg, intramuscular) and maintained, after intubation, by ventilation with O_2_ (100%) mixed with isoflurane (1.5–2.5%). Vital signs including SpO_2_, CO_2_, ECG and heart rate were continuously monitored by a patient monitor (PM-9000 Express, Mindray) during the surgery. Antibiotics and analgesics were used after the surgery.

After the animals were fully recovered, they were trained to fixate a small fixation point on a computer screen for a couple of seconds in exchange for a juice reward, which was delivered through a small tube to the monkey’s mouth by a solenoid valve under computer control [27]. The animals were seated in a custom-made primate chair with their head restrained by fixing the implanted head post to the chair, which was in turn fixed to a rigid frame. Care was taken to maximize animal welfare and minimize suffering. Through visual and social stimulation, the monkeys were provided with enrichment according to National Institutes of Health Guide for the Care and Use of Laboratory Animals to maximize psychological well-being. During the experimental period, they were single housed but had auditory and visual contact with the rest of the colony. They had free access to food but were on controlled fluid access in the housing cage. They earned roughly 80% of their total daily fluid ration during the testing sessions. Out of the experimental period or during the weekends, the monkeys had free access to food and water. The monkeys’ weight and general health were monitored daily. After all experiments in this and other studies were finished, the head post was removed using surgical procedures similar to those for implantation, and the animals were retired to their colony.

Twenty six undergraduate students (10 males and 16 females, mean age ± SEM = 20.3±0.6 years) with normal visual acuity participated in this study. The human experiments were conducted at the University of Lincoln. The Ethical Committee in the School of Psychology, University of Lincoln, approved this study. Written informed consent was obtained from each participant prior to testing, and all procedures complied with the British Psychological Society “Code of Ethics and Conduct” and the World Medical Association Helsinki Declaration as revised in October 2008.

### Stimuli and Apparatus

Digitized images were presented through a ViSaGe graphics system (Cambridge Research Systems) and displayed on a gamma-corrected colour monitor (Mitsubishi Diamond Pro 2070SB for human experiments; Iiyama Vision Master Pro 514 for monkey experiments) with a resolution of 1024×768 pixels and frame rate of 100 Hz. The viewing distance was 57 cm and 100 cm for human and monkey experiments respectively.

**Figure 1:**
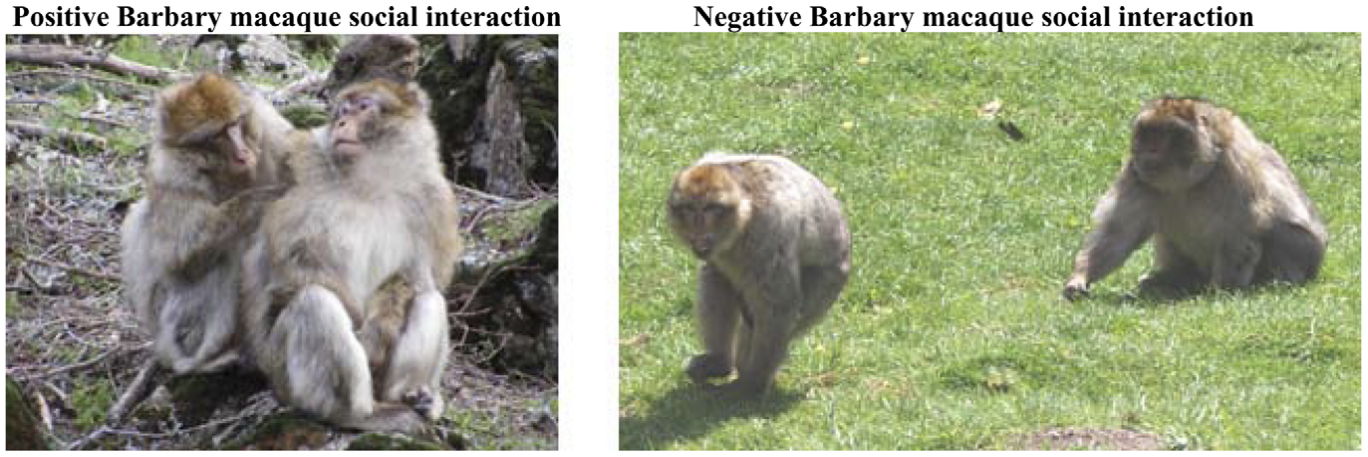
Example of Barbary macaque social interaction scenes.

Colour photographs of dyadic social interactions of five different species (rhesus macaques, Barbary macaques, baboons, lions and humans) were sampled from the internet or the authors’ collections. Each photograph represented either a positive (i.e. affiliation: two individuals grooming, embracing or in social contact with one another) or negative (i.e. aggression: an individual being aggressed by another) social interaction, and consisted of two ‘social roles’, a giver and a receiver of the relevant behaviour (see Fig. 1 for an example). In total, four positive and four negative images from each of the five species were used as stimuli. To control for directional scanning bias (e.g. left gaze bias [7]), stimuli were presented in both their original and mirrored orientation. Given the difficulty to standardize individual’s body size and the distance between two individuals in naturalistic scenes, these images often varied in size. Depending on their width to height ratio, images were consistently fixed to a width of 22.8° (if width was the longer dimension) or a height of 17.1° (if height was the longer dimension). The images were gamma-corrected and displayed at the centre of the screen.

The comparable testing procedure was used for monkey and human subjects. During the recording, the monkeys were seated in a primate chair with their head restrained; the humans sat in a chair with their head supported by a chin rest. All subjects viewed the display binocularly. The horizontal and vertical eye positions of monkey subjects were measured by EyeLink 1000 (SR Research Ltd) with 500 Hz sampling frequency, 0.25–0.5° accuracy and 0.01° root-mean-square resolution; eye positions of human subjects were measured using a Video Eyetracker Toolbox (Cambridge Research Systems) with 250 Hz sampling frequency, 0.125–0.25° accuracy and 0.05° root-mean-square resolution.

To calibrate the eye-tracker a 5-point paradigm was used for monkey subjects. The five points were presented respectively in the center (0, 0), top (0, 7.25°), bottom (0, −7.25°), left (−10.1°, 0) and right (10.1°, 0) of the monitor. A 9-point paradigm was used for human subjects. The nine points were arranged in a 3 × 3 matrix covering the image viewing area. The central point was at the center of the monitor and the distance between adjacent points was 10°. During the calibration, a small fixation point (0.2° diameter, 15 cd/m^2^ luminance) was displayed randomly at one of the 5 or 9 positions across the monitor. The subject was required to follow the fixation point and maintain fixation for 1 s.

After calibrating eye movement signals, a trial was started with a fixation point displayed on the centre of the monitor. If the participant maintained fixation for 1 s, the fixation point disappeared and an image was presented for 10 s. During the free-viewing presentation, the monkeys passively viewed the images, and the humans were instructed to “view the images as you would normally do”. The inter-trial interval was 1 s within which the monkeys received a juice reward without any specific task requirement related to the stimuli.

Each monkey was tested during two sessions separated by at least 48 hours. Each testing session was composed of three consecutive blocks. Within each block, monkeys were randomly presented with eight positive and eight negative (four original and four mirrored) images of each of the five species (total N = 80). Each human participant was tested in a single session. Subjects viewed either the original (17 viewers) or mirrored (9 viewers) images containing four positive and four negative images of each of the five species (total N = 40). After the testing, human participants were asked to categorize each image as either affiliation or aggression in a self-paced free-viewing task. All participants could correctly label the context of social interaction from different animal species.

### Data Analysis

Fixations were extracted from raw eye-tracking data using velocity and duration criteria (lasting longer than 50 ms with less than 0.2° eye displacement at a velocity less than 20°/s [10]). To determine fixation allocation within the image a set of consistent criteria were adopted to divide local regions of different images into: a) the giver or receiver of the affiliation or aggression in each image, b) the background (image area not occupied by the giver or receiver), c) the head and face, or the body region (excluding the head and face) of individuals within each image. See Table 1 for details of the sizes of these regions within different image types (in the majority of cases, the same body region from two individuals within the same image had comparable size). Each fixation was then characterized by its location among local regions and its time of onset relative to the start of the trial. As we required the subjects to fixate a central fixation point prior to image presentation, the first recorded fixation following the image appearance could be interfered with by this central fixation point procedure and was therefore removed from further analysis.

**Table 1:** Proportion (Mean ± SEM) of positive and negative social interaction images occupied by an individual’s body and head/face regions.

Image Species	Body region				Head and face region			
	*Negative*		*Positive*		*Negative*		*Positive*	
	Giver	Receiver	Giver	Receiver	Giver	Receiver	Giver	Receiver
Human	14.97±0.72	16.22±0.20	15.11±0.71	17.00±1.14	1.91±0.15	1.90±0.07	2.71±0.23	3.66±0.34
Rhesus macaque	10.67±0.81	10.55±1.28	21.48±0.26	23.07±0.43	1.97±0.17	1.89±0.22	4.62±0.18	4.49±0.12
Barbary macaque	6.23±0.29	7.21±0.22	16.84±0.82	17.81±0.90	1.71±0.08	1.75±0.09	4.80±0.12	4.77±0.34
Baboon	11.66±0.30	8.46±0.57	20.82±0.64	25.53±0.63	1.96±0.14	1.70±0.07	4.79±0.15	6.16±0.11
Lion	14.87±0.90	11.28±0.43	12.26±0.85	21.23±0.80	4.07±0.42	3.57±0.18	9.97±0.93	5.68±0.38

The number of fixations and viewing time directed at each local region were normalized as a proportion of the total number of fixations and viewing time sampled in that trial. As the same type of local region varied in size across intra- and inter-species images (e.g. two individuals in the same image could vary in body size), the proportion of the image area constituting each local region was subtracted from the proportion of viewing time directed at that region in a given trial. This measure gave us ‘normalized’ viewing time as a percentage for each image region, with positive or negative values indicating more or less viewing time than predicted by a uniform looking strategy [13], [14].

The normalized data were analyzed using a series of generalized linear mixed models (GLMMs) in STATA v10.1 (StataCorp 2007). All the analyses were run using each image presentation as a single data point with subject ID as a random factor to control for the non-independence of the data points [28]. To test whether subjects could differentiate the context of different social interactions, we used GLMMs with Poisson distribution and log link to analyze whether the number of attention shifts between the giver and receiver (i.e. count data) was dependent on the context of the image (i.e. negative or positive). To test whether subjects could differentiate individuals in different social interactions, we used GLMMs with Gaussian error structure and identity link to analyze whether the normalized viewing time was dependent on an individual’s role in the image (i.e. giver or receiver). To test what figure cues were used by subjects to ascertain social role, we used GLMMs to analyze whether viewing time was dependent on the individual’s figure region in the image (i.e. head/face or body). As the same figure region (e.g. animal body) could provide different amounts of information in different social interactions, this analysis was repeated separately on positive and negative images, for each of the five species. For monkey subjects, ‘session ID’ (1–2) and ‘block ID’ (1–3) were control fixed factors in all GLMMs as these variables might affect the subjects’ attention toward stimuli. For human subjects, ‘participant sex’ (male or female) was entered as a control fixed factor as this variable may affect the subjects’ attention toward different sexed stimuli (only male rhesus macaques were tested). GLMM results can be found in Tables 2 to 5.

**Table 2:** Poisson GLMM results for the relationship between the number of attention shifts and image type (i.e. negative or positive) in conspecific and non-conspecific social interaction scenes.

Subject	StimuliSpecies	Negative(mean ± SEM)	Positive(mean ± SEM)	β ± SEM	95% CIs	*Z*	*N*	*P*
Rhesus macaque	Human	3.17±0.18	2.98±0.19	−0.06±0.06	−0.18–0.05	−1.11	383	0.27
	Rhesus macaque	2.68±0.12	3.71±0.18	0.33±0.06	0.22–0.44	5.69	383	<0.001
	Barbary macaque	1.97±0.11	2.75±0.15	0.33±0.07	0.20–0.47	4.95	382	<0.001
	Baboon	2.28±0.11	3.02±0.17	0.28±0.06	0.16–0.41	4.47	384	<0.001
	Lion	2.67±0.14	3.13±0.16	0.16±0.06	0.04–0.27	2.61	384	0.01
Human	Human	3.35±0.17	2.54±0.20	−0.27±0.08	−0.43– −0.11	−3.27	204	<0.001
	Rhesus macaque	2.07±0.15	2.45±0.14	0.17±0.09	−0.01–0.35	1.84	208	0.07
	Barbary macaque	2.23±0.13	2.69±0.16	0.19±0.09	0.01–0.36	2.13	207	0.03
	Baboon	1.86±0.13	2.49±0.16	0.29±0.10	0.10–0.47	3.01	206	0.003
	Lion	2.25±0.15	2.65±0.18	0.17±0.09	−0.01–0.34	1.87	207	0.06

**Table 3:** Linear GLMM results for the relationship between normalised proportion of viewing time and target role (i.e. giver or receiver) in conspecific and non-conspecific social interaction scenes.

Subject	Stimulisocial context	StimuliSpecies	Giver(mean ± SEM)	Receiver(mean ± SEM)	β ± SEM	95% CIs	*Z*	*N*	*P*
Rhesus macaque	Positive	Human	22.22±1.53	7.04±1.50	−15.18±2.11	−19.32– −11.04	−7.19	382	<0.001
		Rhesus macaque	7.12±1.28	18.43±1.61	11.31±2.06	7.21–15.34	5.50	384	<0.001
		Barbary macaque	8.50±1.21	12.46±1.55	3.96±1.95	0.13–7.79	2.03	382	0.04
		Baboon	11.10±1.53	9.32±1.63	−1.77±2.22	−6.13–2.58	−0.80	384	0.42
		Lion	12.31±1.49	12.45±1.62	0.14±2.16	−4.10–4.38	0.07	384	0.95
	Negative	Human	11.98±1.28	20.28±1.38	8.30±1.85	4.67–11.92	4.48	384	<0.001
		Rhesus macaque	14.05±1.34	23.06±1.30	9.01±1.70	5.68–12.34	5.30	382	<0.001
		Barbary macaque	16.75±1.43	18.94±1.36	2.19±1.74	−1.22–5.59	1.26	382	0.21
		Baboon	21.70±1.53	15.34±1.24	−6.37±1.85	−9.99– −2.74	−3.44	384	<0.001
		Lion	22.16±1.52	12.82±1.30	−9.34±1.94	−13.13– −5.55	−4.83	384	<0.001
Human	Positive	Human	36.94±2.89	21.15±2.78	−15.79±4.02	−23.66– −7.91	−3.93	200	<0.001
		Rhesus macaque	19.06±2.17	25.36±2.36	6.30±3.22	0.00–12.60	1.96	208	= 0.05
		Barbary macaque	18.25±1.95	32.27±2.39	14.01±3.08	7.97–20.05	4.55	208	<0.001
		Baboon	15.70±2.32	25.98±2.37	10.27±3.33	3.75–16.80	3.09	206	<0.01
		Lion	35.01±2.57	14.12±2.65	−20.88±3.70	−28.13– −13.63	−5.65	206	<0.001
	Negative	Human	29.02±2.03	34.42±2.95	5.40±2.89	−0.25–11.06	1.87	208	0.06
		Rhesus macaque	22.21±2.06	38.09±2.39	15.88±3.15	9.70–22.05	5.04	208	<0.001
		Barbary macaque	31.73±2.17	41.70±2.24	9.97±3.11	3.87–16.06	3.20	206	<0.01
		Baboon	47.79±2.37	24.39±2.26	−23.40±3.28	−29.83– −16.97	−7.14	206	<0.001
		Lion	35.32±2.51	28.80±2.75	−6.52±3.73	−13.84–0.80	−1.75	208	0.08

**Table 4:** Linear GLMM results for the relationship between normalised proportion of fixations and target role (i.e. giver or receiver) in conspecific and non-conspecific social interaction scenes.

Subject	Stimulisocial context	Stimulispecies	Giver(mean ± SEM)	Receiver(mean ± SEM)	β ± SEM	95% CIs	*Z*	*N*	*P*
Rhesus macaque	Positive	Human	20.75±1.37	6.76±1.36	−14.00±1.91	−17.73– −10.26	−7.35	382	<0.001
		Rhesus macaque	7.85±1.22	18.08±1.50	10.23±1.93	6.45–14.02	5.30	384	<0.001
		Barbary macaque	8.60±1.20	12.28±1.47	3.68±1.88	−0.01–7.37	1.95	382	0.05
		Baboon	11.88±1.52	8.73±1.62	−3.15±2.21	−7.48–1.18	−1.43	384	0.15
		Lion	12.38±1.45	12.61±1.51	0.23±2.06	−3.81–4.26	0.11	384	0.91
	Negative	Human	11.83±1.21	20.00±1.33	8.18±1.77	4.71–11.64	4.62	384	<0.001
		Rhesus macaque	14.21±1.26	22.39±1.18	8.18±1.58	5.10–11.27	5.20	382	<0.001
		Barbary macaque	15.95±1.31	19.00±1.28	3.05±1.57	−0.03–6.12	1.94	382	0.05
		Baboon	21.31±1.43	16.33±1.20	−4.98±1.75	−8.41– −1.56	−2.85	384	<0.01
		Lion	21.34±1.44	12.84±1.25	−8.49±1.84	−12.10– −4.89	−4.62	384	<0.001

**Table 5:** Linear GLMM results for the relationship between normalised proportion of viewing time and target body region (i.e. head/face or body) in conspecific and non-conspecific social interaction scenes.

Subject	Stimulisocial context	Stimulispecies	Head/Face(mean ± SEM)	Body(mean ± SEM)	β ± SEM	95% CIs	*Z*	*N*	*P*
Rhesus macaque	Positive	Human	24.15±1.69	5.11±1.74	−19.04±2.40	23.75– −14.33	−7.92	382	<0.001
		Rhesus macaque	26.85±1.51	−1.30±1.39	−28.15±2.05	−32.17– −24.14	−13.74	384	<0.001
		Barbary macaque	23.14±1.53	−2.18±1.46	−25.32±2.10	−29.44– −21.21	−12.06	382	<0.001
		Baboon	26.11±1.64	5.18±1.72	−20.93±2.36	−25.56– −16.30	−8.86	384	<0.001
		Lion	30.65±1.87	2.01±1.38	−28.64±2.21	−32.98– −24.31	−12.95	384	<0.001
	Negative	Human	15.99±1.36	16.26±1.50	0.27±2.00	−3.66–4.19	0.13	384	0.89
		Rhesus macaque	25.48±1.38	11.62±1.52	−13.86±18.90	−17.58– −10.14	−7.30	382	<0.001
		Barbary macaque	21.43±1.52	14.26±1.44	−7.16±1.88	−10.85– −3.49	−3.82	382	<0.001
		Baboon	15.08±1.18	21.96±1.46	6.88±1.75	3.46–10.31	3.94	384	<0.001
		Lion	18.43±1.39	16.55±1.85	−1.88±2.26	−6.31–2.55	−0.83	284	0.41
Human	Positive	Human	49.41±2.95	8.67±3.30	−40.74±4.43	−49.43– −32.06	−9.19	200	<0.001
		Rhesus macaque	38.89±2.21	5.53±2.18	−33.36±3.11	−39.46– −27.27	−10.73	208	<0.001
		Barbary macaque	49.77±2.71	0.75±2.98	−49.02±4.03	−56.92– −41.11	−12.15	208	<0.001
		Baboon	64.68±2.10	−23.00±2.19	−87.67±3.04	−93.64– −81.71	−28.82	206	<0.001
		Lion	67.31±1.84	−18.17±1.71	−85.48±2.52	−90.42– −80.55	−33.94	206	<0.001
	Negative	Human	40.76±2.65	22.68±2.59	−18.08±3.72	−25.37– −10.80	−4.87	208	<0.001
		Rhesus macaque	47.42±2.25	12.87±2.78	−34.55±3.57	−41.55– −27.55	−9.67	208	<0.001
		Barbary macaque	48.67±2.57	24.76±2.78	−23.91±3.78	−31.32– −16.50	−6.32	206	<0.001
		Baboon	68.52±2.42	3.66±2.31	−64.85±3.35	−71.41– −58.29	−19.38	206	<0.001
		Lion	57.07±2.39	7.05±2.70	−50.02±3.62	−57.11– −42.94	−13.83	208	<0.001

## Results

Across all images, rhesus subjects spent significantly more time viewing images of their own species (on average 36% of 10 s image presentation time) compared to images of Barbary macaques (29%), baboons (29%), lions (30%) and humans (31%; all comparisons conspecific versus other species images: GLMM, *p values*<0.001). The social context of the scene, on the other hand, did not affect their viewing time (positive images = 30%, negative images = 31%; GLMM, *p* = 0.47) but had an impact on the rate of their attention shifts between the giver and receiver in the images (Table 2). Compared to negative images, rhesus subjects tended to look back and forth between individuals more frequently when viewing positive interactions between rhesus macaques, Barbary macaques, baboons and lions. There was no significant difference in the number of attention shifts when rhesus subjects viewed positive and negative human images.

### 1) Can Rhesus Macaques Differentiate between Conspecific Individuals Based on their Roles in Different Social Interactions?

**Figure 2:**
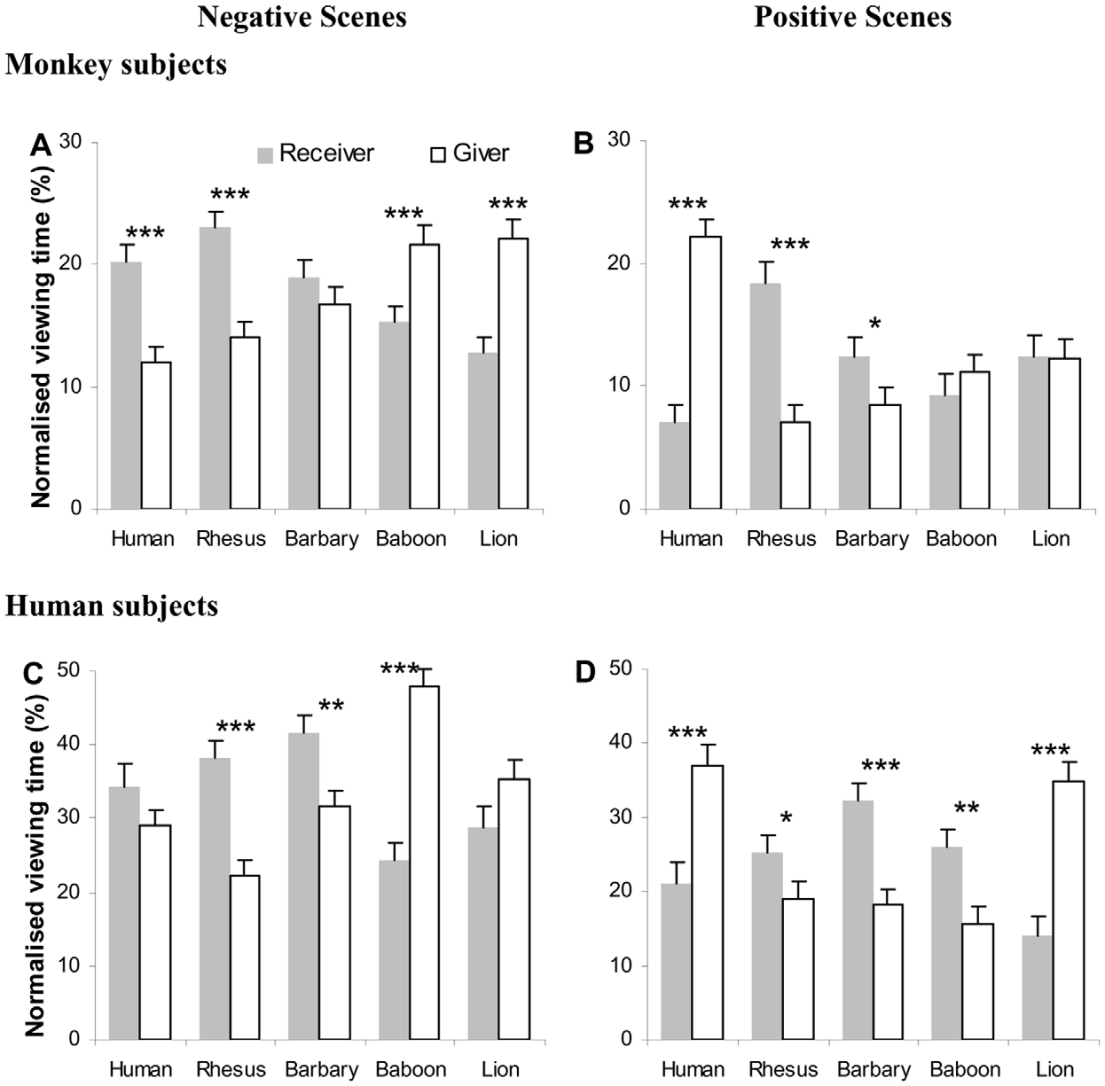
Proportion of normalized viewing time directed at the giver and receiver in negative (A and C) or positive (B and D) social interaction scenes between conspecifics or non-conspecifics. Error bars represent SEM. * *p*<0.05, ** *p*<0.01, *** *p*<0.001.

The comparison of normalized viewing time directed at the two individuals within an image indicated that rhesus subjects could differentiate the roles of conspecific individuals in images of different social contexts (Fig. 2, Table 3). Specifically, they spent proportionally more time viewing the receiver than the giver in both positive (18% on the groomee vs. 7% on the groomer) and negative (23% on the victim vs. 14% on the aggressor) rhesus macaque images (also see Table 4 for normalized fixation distribution which was closely correlated with viewing time distribution). This gaze preference towards the receiver, however, was not consistent when viewing social interactions of non-conspecifics. Instead, the role of the most viewed individual within an image was species- and context-dependent.

### 2) Do Rhesus Macaques Generalise their Gaze Behaviour Across Non-conspecific Social Interactions?

For the negative non-conspecific social interaction scenes, rhesus subjects displayed the same viewing behaviour towards humans as they did towards conspecific images, with more gaze at the receiver than the giver. However, they directed an indistinguishable amount of gaze at the two individuals in Barbary macaque images, and viewed longer at the giver than the receiver in baboon and lion images (Fig. 2A). Interestingly, their viewing behaviour towards non-human images seemed to be correlated with the phylogenetic distance from the viewed species. The difference in viewing time allocated at the receiver and the giver was 9% for rhesus macaques, 2% for Barbary macaques, −6% for Baboons and −9% for lion images.

For the positive non-conspecific social interaction scenes, rhesus subjects inspected longer at the receiver than the giver in Barbary macaque images, similar to the viewing of their own species. However, they spent an equal amount of time viewing two individuals in both baboon and lion images, and directed significantly more gaze at the giver than the receiver in human images (Fig. 2B). Similarly to the negative scenes, the viewing behaviour towards non-human positive scenes also changed with the phylogenetic distance from rhesus subjects. The difference in viewing time allocated at the receiver and the giver was 11% for rhesus macaques, 4% for Barbary macaques, and did not significantly differentiate from 0 for baboon and lion images. However, unlike negative human images (20% time on the receiver vs. 12% on the giver), which attracted the same viewing pattern as rhesus macaque images, positive human images induced an opposite gaze pattern, with the giver receiving more inspections (7% on the receiver vs. 22% on the giver). In summary, the amount of viewing time directed at an individual within social interaction scenes was role-, species- and context-dependent, implying that rhesus subjects can differentiate different types of social interaction (at least from those closely-related species).

### 3) Which Local Regions do Rhesus Macaques Attend More Frequently to Extract Diagnostic Visual Cues for Processing Scene Contents?

**Figure 3:**
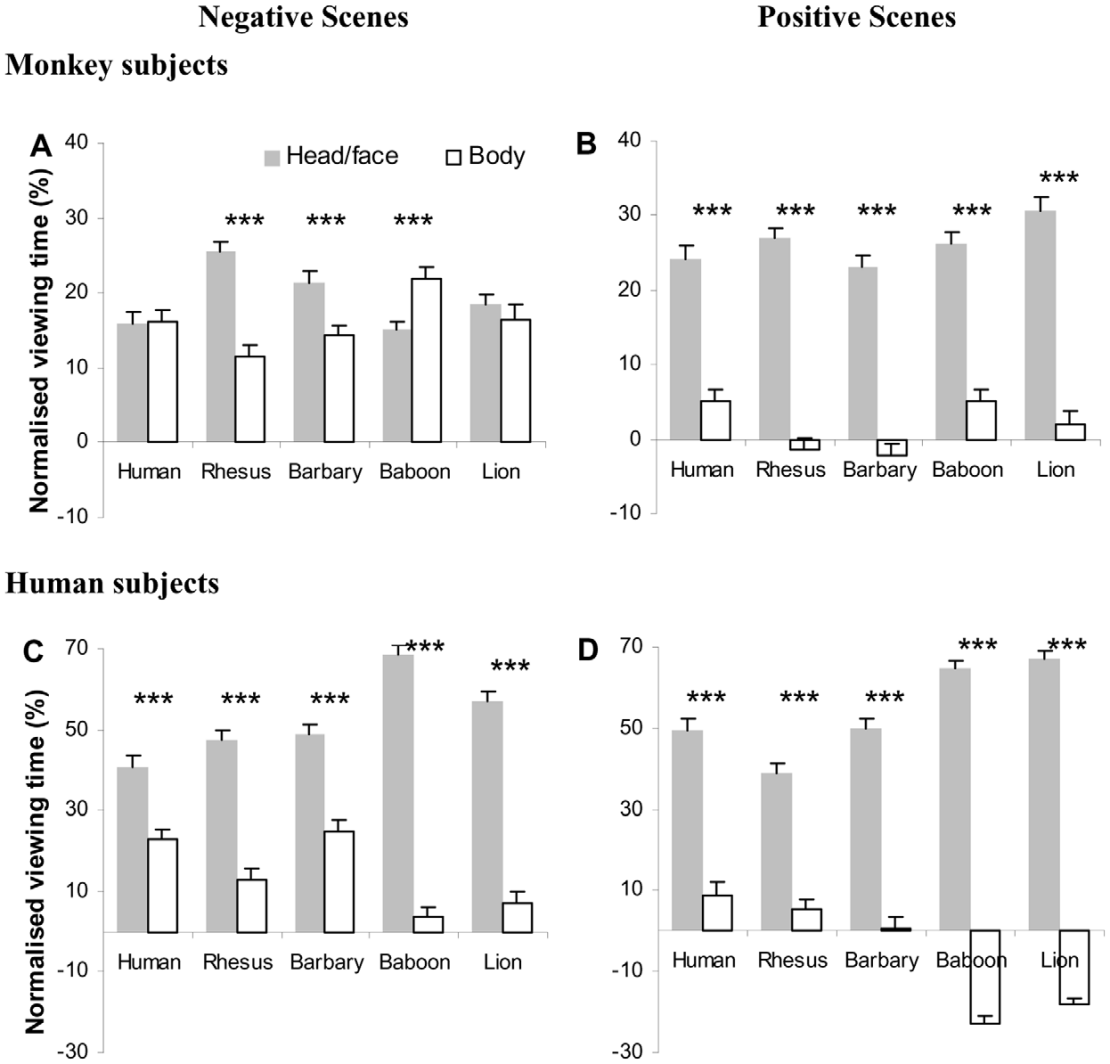
Proportion of normalized viewing time directed at the head/face and body region while inspecting individuals in negative (A and C) or positive (B and D) social interaction scenes between conspecifics or non-conspecifics. Error bars represent SEM. * *p*<0.05, ** *p*<0.01, *** *p*<0.001.

Rhesus subjects spent proportionally more time viewing the head and face region, compared to the rest of the body, in the majority of cases (Table 5; Fig. 3A and 3B), suggesting a stereotypical gaze pattern of frequent inspection towards the face/head region when observing social interactions. Quantitatively, the amount of viewing time directed at the face/head and body region was also species- and context-dependent. The monkeys inspected the body region for longer when viewing negative images, compared to positive, regardless of the viewed species (compare empty bars in Fig. 3A with 3B). In fact, when viewing positive images of all species, the majority of viewing time was at the head/face region and very little at the body region. When viewing the negative images, the monkeys viewed longer at the head/face of the rhesus and Barbary macaques, but shorter at the baboons’ head/face. The head/face and body regions in lions and humans, on the other hand, attracted the same proportion of viewing time. Taken together, these results suggest that different figure regions provide different diagnostic cues for the interpretation of different types of social interaction.

### 4) Do Rhesus Macaques Share Similar Viewing Behaviour to Humans in the Viewing of the Same Social Scenes?

The rhesus subjects on average spent 3.1 s (including time for fixations and saccades) out of 10 s trial duration to inspect the presented images, which was shorter than humans during the 10 s of image presentation. To make the gaze patterns comparable between human and monkey subjects, we only analyzed human gaze distribution for the first 3.1 s of image inspecting time per trial. Consistent with previous reports that monkeys tend to explore the spatial extent of the natural scene more thoroughly [20] and scan the background scene (image area not occupied by humans/animals) more often than humans [21], we observed that human’s gaze was much more concentrated on the humans/animals while viewing the same set of social interaction scenes. The normalized viewing time directed at the figures (receiver+giver) within each image was 58% and 30% for human and monkey subjects respectively.

Like monkey subjects, human subjects made or tended to make more attention shifts between the giver and receiver when viewing positive, compared to negative, interactions between rhesus macaques, Barbary macaques, baboons and lions. Unlike monkey subjects, human subjects made significantly more attention shifts when viewing negative human images than positive ones (Table 2).

The pattern of gaze allocation towards the two individuals within a social interaction image, however, was strikingly similar between rhesus and human subjects (Fig. 2, Table 3). Indeed, the negative images from a given species elicited qualitatively identical gaze distribution from rhesus and human subjects (compare Fig. 2A with 2C): both species tended to inspect longer at the receiver in rhesus macaque, Barbary macaque and human images, but shorter at the receiver in baboon and lion images. The positive images elicited a somewhat less consistent gaze distribution from rhesus and human subjects (compare Fig. 2B with 2D). On the one hand, both species spent longer viewing the receiver in rhesus and Barbary macaque images, and shorter at the receiver in human images. On the other hand, while rhesus subjects spent an equal amount of time viewing the two individuals in baboon and lion images, humans directed more gaze at the receiver in baboon images, and more at the giver in lion images.

The gaze distribution within different figure regions was also remarkably similar between rhesus and human subjects (Fig. 3, Table 5). Both species tended to view longer at the head/face than the body region when inspecting individuals (especially in positive scenes), and gazed more at the body in negative interactions compared to positive interactions. There were, however, some quantitative differences between the two viewer species. Specifically, humans viewed the head/face significantly longer than the body region regardless of the nature of the social interaction. Such allocation difference in viewing time at the head/face and body region was more evident when inspecting baboons and lions. The rhesus subjects, on the other hand, directed similar or even slightly higher proportion of viewing time at the body in comparison with the head/face region when inspecting negative interactions in baboons, lions and humans, implying a gaze strategy difference in detecting threatening cues between human and monkey observers. Overall, in spite of some small or quantitative differences, our results (Figs. 2 and 3) point towards largely overlapping gaze strategies between monkeys and humans in their visual analysis of social interactions.

## Discussion

The capacity to discriminate biologically relevant social stimuli based on their relevance to individual fitness is likely to be under selective pressure. From an evolutionary perspective, primate social attention should be guided by selectively analyzing the most informative cues associated with social interactions and behaviours. For example, when presented with images of familiar monkeys of different dominance status, male rhesus macaques needed above-average juice reward to view monkey faces of subordinates, but were willing to sacrifice fluid to view faces of dominant males [29]. This is because a dominant animal represents both a potential threat and a more valuable social partner (e.g. in terms of agonistic support) than subordinates, and thus a more relevant target of visual attention. In the current study, with naturalistic social interaction scenes, we found that rhesus monkeys could spontaneously discriminate individual conspecifics based on their roles in social interactions. Between the two characters within a scene, they gazed more at the receiver than the giver in both negative and positive scenes.

Such discrimination between social roles may involve different social cognition processes. In the negative social interactions, the more frequent looking at the receiver (i.e. the victim) could be due to three inter-dependent processes: avoidance towards the aggressor (i.e. the giver), gaining of social benefits through observation and/or empathy towards the victim. Firstly, eye contact is a threatening display in macaques [24]. In fact, male rhesus monkeys produce appeasement gestures and avoid gaze contact in response to videos of threatening males [22], [23]. Secondly, focusing on the identity and behaviour of the victim of aggression may give social benefits to the animal observing an agonistic interaction between conspecifics. For example, victims of aggression are more likely to give grooming to a bystander in the aftermath of a conflict than when they have received no aggression [30]. Moreover, the ‘loser effect’ predicts that victims of aggression who have lost a fight tend to lose fights again in the future, either with the former opponent or with other group members, other things being equal (e.g. agonistic support) [31], [32]. As such, by attending agonistic interactions monkeys can gather information on which animal to aggressively target to raise or maintain their rank position, and/or to coerce for grooming opportunities [30], [33]. Thirdly, showing empathy or concerns for others in distress is evident in humans (even in two-year-old infants [34]) and might also exist in non-human primates [35], [36]. Although the role of empathy here is speculative, the former two processes described above can lead to relatively longer viewing at the receiver in negative scenes. The receiver in the positive interactions, on the other hand, represents the groomee in our images. Dominant animals receive more grooming than subordinates in a range of primate species, including rhesus macaques [37]. Frequent looking at the receiver may reflect the viewer’s intention to attend to high-status individuals as they have a higher impact on the viewer’s own behaviour [29]. Additionally, focusing on the receiver of grooming is beneficial for the occurrence of generalized reciprocity, as the receiver is the individual more likely to give grooming to a third animal later (e.g. the one attending to the grooming interaction [4]).

To what extent can this social role-sensitive gaze behaviour in rhesus macaques be relatively attributed to experience or phylogeny? The comparison of their gaze distribution at conspecific and non-conspecific interactions provided some insights. Although these laboratory-raised rhesus monkeys had frequent interactions with conspecifics and human carers/researchers, their viewing patterns toward rhesus macaque and human images were context- and species-dependent. For the negative interactions, they gazed longer at the receiver in both rhesus macaque and human images, suggesting the adoption of a similar social attention pattern. For the positive interactions, they looked more at the conspecific receiver but more at the human giver, suggesting the adaptation of gaze behaviour according to their social contact experience.

Interestingly, the monkey’s gaze distribution in viewing unfamiliar non-conspecific images was systematically varied with their phylogenetic distance from the viewed species. The more phylogenetically distant the taxon (i.e. from Barbary macaque, belonging to the same genus; to baboon, same order; and lion, same class), the less the monkeys attended at the victim and the more at the aggressor in the negative interactions (Fig. 2A); implying a shift of gaze pattern (from displaying empathy to examining threat) that may be phylogenetically-based. For the positive interactions (Fig. 2B), monkeys attended to the groomee (with the same viewing pattern displayed for conspecific images but with decreased viewing time) in Barbary macaques but did not differentiate between groomee and groomer in baboons and lions. Given that the studied animals have never encountered species other than conspecifics and humans, it seems that their social attention to interactions (especially negative interactions) between unfamiliar species is strongly influenced by innate bias. In other words, the more phylogenetically distant a species is, the less relevant their social interactions become. This is possibly due to different species-specific facial displays and body postures in different social contexts.

Being the major animal model of human visual perception, rhesus monkeys have a close evolutionary connection with humans in the neuroanatomical organization of the visual system, as well as in visual and social behaviours. Earlier comparative studies have found a remarkable similarity in gaze patterns between rhesus monkeys and humans when exploring face images, natural scenes and movie clips [7]–[15], [20], [21]. Here we extend this similarity to the processing of more complex, naturalistic social interactions, in which both species demonstrated a *qualitatively* similar role-sensitive, species- and context-dependent gaze distribution (especially when inspecting negative social images, Fig. 2). Both species also attended to the same local figure region to extract informative social cues. They tended to gaze more at the face than the body region, and inspect relatively longer at the body region in negative relative to positive social interactions (Fig. 3). Taken together, the current study suggests that monkeys and humans share a homologous social attention strategy when processing social scenes.

However, notable differences between human and monkey observers in viewing conspecific and non-conspecific social scenes can still be identified. Unlike monkey viewers, the quantitative difference in viewing time directed at the receiver and the giver from human viewers was not systematically influenced by the viewed species in negative interactions (Fig. 2C), and was significantly different when viewing baboons and lions in positive interactions (Fig. 2D). These differences could be due to the fact that humans have acquired knowledge about different non-conspecific animals through various sources, which could bias their gaze behaviour. Nonetheless, the capacity to perform social evaluation through the observation of a conspecific’s social behaviour, emerges very early in human development. Even three-month old infants prefer individual characters behaving prosocially to those behaving antisocially in various social scenarios [38]. It seems that attending to and evaluating individuals based on their mutual treatment is fundamental to perceive the social world, and such capability could be largely influenced by innate bias.

Furthermore, humans spent more time focusing on the individuals in the image, especially on the face region, than monkey viewers when examining both conspecific and non-conspecific images (Fig. 3). Similar differences in gaze behaviour also exist between human and chimpanzee viewers [39] which could be related to species-specific forms of social interaction. In monkey and chimpanzee societies, where long fixation towards a conspecific face represents a strong signal of threat [25], viewers may simply look at the face briefly to reduce direct gaze contact, especially when inspecting figures in negative social interactions.

Taken together, our findings revealed that monkey and human observers adopt role-sensitive, species- and context-dependent gaze behaviour when inspecting conspecific and non-conspecific social interactions, which is qualitatively similar but with some marked quantitative differences between the two species. This suggests that social attention in rhesus monkeys and humans share some basic innate properties and mechanisms, but is also modulated by experience. Thus, social attention is likely to be a biological adaptation in these two species.

However, it should be noted that some types of social interaction, such as aggression, could contain similar subject action (e.g. chasing/running action) and image cues (e.g. motion and distance cues) across many different species. These inherent scene properties could partially drive the viewer’s gaze allocation. Therefore, it is possible that the very basic properties of social attention may be similar across all primate, as well as non-primate species. Given we presented snapshots of social interactions on a computer screen in this study, it also remains a question to what extent the monkey viewers would interpret such scenes as ecologically relevant. Moreover, different social interaction scenes often differ in image properties. For instance, the distance between two individuals in an aggression scene (e.g. one animal chasing another) is likely to be larger than in an affiliation scene (e.g. one animal grooming another), and an aggression scene may contain more movement cues than an affiliation scene. Although many of these variables are inherent properties of the scene and could be essential to define the scene’s social context, the gaze allocation to different individuals and/or different body regions in the image could be partially affected by these image variables. Future studies could systematically manipulate these variables (e.g. varying distance between two individuals in the image without changing its social context) to examine to what extent they affect gaze behaviour when viewing social interaction scenes.
